# The efficacy of bracing in the treatment of progressive early-onset scoliosis

**DOI:** 10.1038/s41598-024-61030-5

**Published:** 2024-05-03

**Authors:** Haixia Li, Jigong Wu, Lizhi Song, Shuilin Shao, Zhiming Chen, Jiaxu Wang, Bo Gao, Litao Huo

**Affiliations:** 1 Department of Spine Surgery, Strategic Support Force Medical Center, Beijing, 100101 China; 2 Beijing Lizhi Rehabilitation Aids Center, Beijing, 102208 China

**Keywords:** Progressive early-onset scoliosis, Nonsurgical, Scoliosis, Bracing, Delaying surgery, Rib-vertebral angle difference, Outcomes research, Paediatric research

## Abstract

Serial casting as one of the applications to treat early-onset scoliosis has been reported efficiently to improve deformity, but no report has focused on the efficacy of braces in the treatment of congenital early-onset scoliosis and comparison with progressive idiopathic early-onset scoliosis. Patients with progressive EOS treated with braces in our institution with a minimum of 4 years follow-up were reviewed. Two groups according to the etiological diagnosis were analyzed and compared: the congenital scoliosis (CS) group and idiopathic scoliosis (IS) group. The success cases and the failure cases were also compared. 27 patients with an average main Cobb angle of 38.19° (20–55) underwent initial bracing at an average age of 55.7 months (24–108), the average follow-up time was 76.19 months (49–117). In IS group the main Cobb angle was corrected to 18.69 ± 12.06° (48.61%) following the first bracing; the final Cobb angle was 23.08 ± 22.15°(38.76%) after brace removal. In CS group the main Cobb angle was corrected to 33.93 ± 10.31°(17.1%) following the first bracing and 37.93 ± 14.74°(3.53%) after brace removal. Both coronal chest width and T1-T12 height increased dramatically from pre-bracing to the last follow-up. Patients diagnosed as IS tended to have a better result in main Cobb angle correction than that of CS (*P* = 0.049). By the time of last follow-up, 8 patients had undergone surgery, and the operation time was postponed by 68.88 ± 26.43 months. For patients with progressive early-onset scoliosis, bracing is an efficient nonsurgical alternative to casting, and some of them can be cured; if not, eventual surgical intervention can be delayed for a period of time without restrictions on the thoracic cavity.

## Introduction

Scoliosis diagnosed before 10 years old is known as early-onset scoliosis (EOS). Congenital scoliosis and some Idiopathic scoliosis with certain characteristics can be considered as a progressive scoliosis^1–4^. These characteristics include Cobb angle^3^20°, and rib vertebral angle difference (RVAD) >20°, or phase II rib-vertebra relationship. The treatment of progressive EOS that tends to cause severe deformity is a steep challenge for orthopedic surgeons. Treatment scoliosis on the early-onset stage can control the curve progression, preserving the growth potential until the patient is of appropriate age or size for surgery, or even avoiding surgery.

The treatments for EOS include observation, nonsurgical techniques, and surgical intervention. Observation may be appropriate for resolving curves with good prognostic indicators, such as RVAD < 20°, age less than 1 year, Cobb angle < 20°, and no curve progression^1–3^. Nonsurgical techniques such as bracing in idiopathic EOS with smaller curves, serial casting for progressive EOS, a transition toward the use of operations, such as growing rods (GR), vertical expandable prosthetic titanium rib (VEPTR) techniques, and the Shilla technique in moderate-to-severe scoliosis have occurred over the past decades, but they continue to be challenging with high complication rates^5–9^. In addition to complications from surgery, the neurodevelopmental implications of repeated anesthesia in infants should be considered^10^.

Serial casting as a nonsurgical application for infantile progressive EOS has been reported with satisfactory results^11–15^. However, it has been reported in the literature that anesthesia with tracheal intubation is needed for plaster orthopedics, which has neurodevelopmental implications, and the cast is applied with a specific table with gentle in-line traction, which is inconvenient^14^,^16^,^17^. In addition, traction after anesthesia has the risk of spinal cord injury. In patients with underlying pulmonary disease, the casting process may induce respiratory complications, and casting results in an increased peak inspiratory pressure (PIP) due to transient restrictive pulmonary processes; after windows are cut out, the PIP is reduced but not to baseline^18^. Bracing has been certified in the treatment of adolescent idiopathic scoliosis, but evidence supporting the efficacy of braces in the treatment of congenital early-onset scoliosis and comparison with progressive idiopathic early-onset scoliosis is lacking. We wished to analyze the results of bracing for this type of patient at our center and provide a new method to correct deformities or delay surgical intervention.

## Materials and methods

### Patient characteristics

Institutional Review Board approval of Strategic Support Force Medical Center was obtained for this study. Patients with progressive EOS treated with braces in our institution between June 2011 and July 2023 who met the following criteria were included. (1) The age of initial bracing was no more than 10 years old; (2) the minimum follow-up time was 4 years; (3) etiological diagnosis of congenital scoliosis (CS), idiopathic scoliosis (IS) with Cobb angle of 25° or more, or Cobb angle of no less than 20° with RVAD > 20°, or phase II rib-vertebra relationship; (4) patients never treated with spinal deformity surgery. Patients of nonprogressive idiopathic scoliosis, neuromuscular scoliosis and EOS of Syndromic scoliosis, such as Marfan syndrome, Klippel-Feil syndrome, etc., were excluded. Consistent with Scoliosis Research Society(SRS) criteria^19^, we defined improvement to be a decrease of more than 5° of the Cobb angle from brace initiation to the final control, stabilization to be a Cobb angle variation ± 5°, and failure to be an increase of more than 5°, Cobb > 45° at last control or at maturity, or a final need for surgery. Improved and stabilized patients were included in a simple success group. Thus, treatment outcome could be classified as success or failure.

### Methods

All methods were performed in accordance with the relevant guidelines and regulations. Informed consent was obtained from all the legal guardian(s) of the participants. Patients with progressive EOS treated with braces in our institution with a minimum of 4 years follow-up were reviewed. Two groups according to the etiological diagnosis were analyzed and compared: the congenital scoliosis (CS) group and idiopathic scoliosis (IS) group. In addition, the success group and the failure group were also compared. Demographic and clinical information included sex, age at initial bracing and the most recent follow-up, months of follow-up and months of treatment. All patients had anteroposterior radiographs, and most of them had lateral radiographs of the spine before the initial brace and after the brace. To avoid the potential confounding of residual within brace, all braces were removed 24 h before radiographs examination. The location of the apical vertebra and total vertebral segments in the major curve were recorded, and the major curve magnitude (Cobb angle), proximal and distal compensatory curve, coronal chest width (measured by drawing a horizontal line between the inner edge of 1 rib to the inner edge of the opposite rib at the widest point of the rib cage)^20^ and T1-T12 height (measured from the middle of the upper endplate of T1 to the middle of the lower endplate of T12) were measured at pretreatment, immediately after initial bracing, and at the last follow-up. Measurements of thoracic kyphosis and lumbar lordosis were not all measured due to not be able to obtain lateral radiographs of the spine for all patients, on account of most of them did not have a large sagittal plane deformity. No complications were recorded during the treatment. Final outcomes at the time of last follow-up were collected. All measurements were taken by a single author with both interobserver and intraobserver reliability measurements performed by a second observer. Brace removal was left to the surgeon’s discretion when the curve resolved and believed to be stable enough. No data was collected to monitor brace compliance.

### Brace technique

Cheneau brace was used in our cases. Based on the traditional brace, multipoint translational force focused on the apex of the curve, and detorsional forces reaching the best possible frontal and sagittal alignment were performed according to the radiographs of the spine in our brace. In addition to the treatment of scoliosis, it can reduce the impact on patients' lung function and improve their quality of life during treatment. The initial wearing time of the brace was 22 h/d. Patients were followed up every 3–6 months. Interval X-rays were occasionally obtained during the follow-up, and the brace wearing time was adjusted according to the growth of the patient and the changes in the Cobb angle. If the deformity was corrected satisfactorily and remained stable for 6 months, the wearing time could be reduced to 18 h/d; otherwise, the wearing time of the brace should be kept at 22 h/d.

### Statistical analysis

Statistical analysis was conducted using SPSS statistical software (version 20, IBM, Inc., Armonk, NY). Descriptive results are expressed as mean ± standard deviation. Differences in categorical data between success and failure groups and that between IS and CS groups were compared using the Chi-square test and Fisher’s exact test, and descriptive results were compared using the independent-sample t-test. Differences between pretreatment and first in brace, pretreatment and last follow-up were investigated with the paired sample t-test. A statistically significant difference was defined as P < 0.050.

## Results

27 progressive EOS patients (female vs. male: 16 vs. 11) met all the inclusion criteria. Patient demographic characteristics, radiographs, and final outcomes were collected. The average age at first bracing was 55.70 months (24–108), and patients were followed up for 76.19 months (49–117). The average number of segments included in the main curve was 6.07 (3–9), 12 cases had the apical vertebra located in the thoracolumbar or lumbar region (44.4%), and 4 patients had segmental kyphosis (14.81%). Table 1.

**Table 1:** Pretreatment characteristics of all patients.

Patients’ characters	Average (range)/count (percentage)
**Demographic**	
Female: Male	16:11
CS: IS [n (%)]	14:13
Age at initiation of bracing (months)	55.70 (24–108)
Age at last follow-up, months	130.26 (87–165)
Wearing time, months	71.93 (34–117)
Follow-up duration, months	76.19 (49–117)
**Radiographic**	
Cobb angle, °	38.19 (20–55)
Thoracic kyphosis , °	22.04 (5–49)
Lumbar lordosis, °	50.72 (32–74)
Coronal chest width, mm	168.39 (133.72–197.08)
T1-T12 height, mm	171.41 (136.47–209.43)
Segments included in main curve	6.07 (3–9)
Apical vertebra of thoracolumbar or lumbar [n (%)]	12 (44.4)
With segmental kyphosis [n (%)]	4 (14.81)

CS: congenital scoliosis; IS: idiopathic scoliosis.

**Figure 1:**
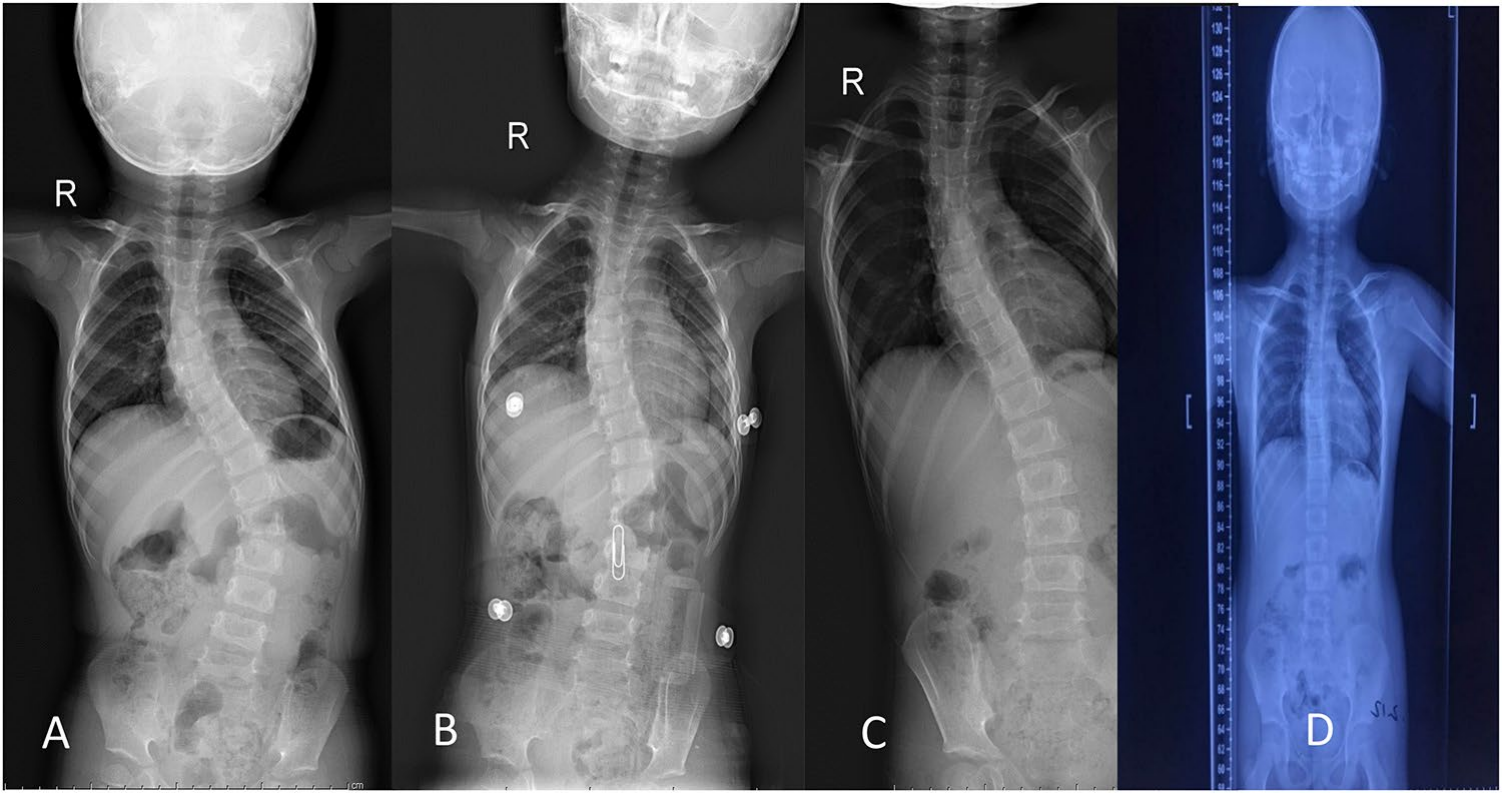
A 31-month-old boy diagnosed with idiopathic EOS with a thoracolumbar apical vertebra. X-ray obtained before brace placement (**A**), after initial brace placement (**B**), at the 2-year follow-up (**C**) and at the last follow-up (**D**).

Up to the final follow-up, 8 patients had resolved and stopped bracing but were still under observation in case of regression (Fig. 1). 8 patients had undergone surgery, and the average postponement time was 68.88 ± 26.43 months. 11 patients (40.74%) were still under a bracing regime at the last control, including 9 success cases and 2 failure cases. No complications related to bracing were noted in the clinical chart.

The major curve Cobb angle before bracing was 38.19 ± 10.13°, which was corrected to 26.59 ± 3.43°(*P* = 0.000) in an initial bracing and 31.26 ± 19.53°(*P* = 0.054) at the final follow-up. Coronal chest width and T1-T12 height were measured in all 27 patients, and they were all increased dramatically from initial to last follow-up (*P* = 0.000) (Table 2).

**Table 2:** The radiological change between pretreatment and first in brace (P1) and that between pretreatment and last follow-up (P2) were analyzed by paired-samples T test.

Radiographic characteristics	Pretreatment	First in brace	Last follow up	p1	p2
Cobb angle of main curve,°	38.19 ± 10.13	26.59 ± 3.43	31.26 ± 19.53	0.000	0.054
Coronal chest width, mm	168.39 ± 17.59	168.45 ± 16.77	203.94 ± 25.23	0.961	0.000
T1-12 height, mm	171.41 ± 20.78	177.67 ± 23.55	222.29 ± 32.80	0.000	0.000
Proximal compensatory curve,°	16.35 ± 11.91	12.23 ± 11.51	17.78 ± 15.56	0.008	0 0.631
Distal compensatory curve,°	24.00 ± 11.02	20.44 ± 11.80	17.50 ± 12.20	0.044	0.010
Thoracic kyphosis,°	21.56 ± 12.61	18.94 ± 12.45	25.04 ± 13.82	0.106	0.172
Lumbar lordosis,°	50.78 ± 12.97	47.61 ± 13.75	46.64 ± 16.34	0.148	0.128
Absolute value of T1-CSVL, mm	13.76 ± 10.15	14.33 ± 12.25	12.87 ± 11.36	0.829	0.781
Absolute value of SVA, mm	17.17 ± 22.12	26.45 ± 15.89	28.04 ± 16.22	0.134	0.095

CSVL: cervical 7 to center sacral vertical line; SVA: sagittal vertebral axis.

We analyzed and compared the success group and failure group. A total of 62.96% of the patients met the success criteria at the last follow-up. Sex, age at initial bracing and Follow-up duration, age at last follow-up, wearing time, main Cobb angle, thoracic kyphosis, lumbar lordosis, coronal chest width, T1-T12 height, and segments included in the main curve were not significantly different between the two groups (P > 0.05). Patients diagnosed with IS tended to have a good result, but the difference was not significant (*P* = 0.695), and those with the apical vertebra located in the thoracolumbar or lumbar region also achieved better improvement (9 success and 3 failure, *P* = 0.424). The correction rate of the initial brace was 38.64% ± 28.08 in the success group and 21.44% ± 17.14 in the failure group (*P* = 0.093) and that of the last follow-up was 47.89% ± 31.62 in the success group and -26.08% ± 37.84 in the failure group (*P* = 0.000). (Table 3).

**Table 3:** Radiographic and treatment parameters: success versus failure.

Patients’ characters	Success (n = 17)	Failure (n = 10)	*P* value
Pretreatment Cobb angle,° (SD)	37.00 (9.44)	40.2 (11.43)	0.439
Cobb angle of initial brace,° (SD)	23.29 (13.19)	32.2 (12.51)	0.097
Correction rate of initial brace,%(SD)	38.64 (28.08)	21.44 (17.14)	0.093
Cobb angle of last follow up,° (SD)	20.12 (13.75)	48.9 (14.76)	0.000
Correction rate of last follow up,%(SD)	47.89 (35.21)	-26.08 (37.84)	0.000
Thoracic kyphosis,° (SD)	23.73 (12.61)	19.50(10.97)	0.396
Lumbar lordosis,° (SD)	48.53 (7.50)	54.00 (15.99)	0.333
Coronal chest width, mm(SD)	166.79 (17.54)	171.11 (18.27)	0.549
T1-T12 Height, mm(SD)	171.76 (21.80)	170.80 (20.04)	0.909
Segments included in main curve, n(SD)	5.88 (1.50)	6.40 (1.78)	0.425
Apical vertebra of thoracolumbar or lumbar, [n (%)]	9 (75)	3 (25)	0.424
Female, [n (%)]	10 (62.5)	6 (37.5)	1.000
Diagnosis of IS, [n (%)]	9 (69.23)	4 (30.77)	0.695
Age at initial bracing, Months(SD)	53.12 (23.93)	59.30 (21.93)	0.821
Wearing time, Months(SD)	72.41 (18.84)	71.10 (24.07)	0.876
Follow-up duration, Months(SD)	73.65 (18.37)	80.50 (18.17)	0.356
Age at last follow-up, Months(SD)	126.00 (23.66)	137.30 (18.47)	0.208

Patients were also grouped based on etiology diagnosis; 14 patients had an associated congenital spinal anomaly of vertebral deformity and were diagnosed with congenital scoliosis (CS), 7 patients had hemivertebra, and 7 patients had wedge vertebra, one of which had a tethered spinal cord detethered by surgery before bracing, 13 patients was progressive idiopathic scoliosis (IS). The pretreatment main Cobb angle in the two groups was similar (*P* = 0.108), there was a significant difference in the main curve correction rate in the first bracing [48.61% ± 23.66 (IS) vs. 17.10% ± 16.89 (CS), *P* = 0.000] and a not significant difference in that of the last follow-up [38.76% ± 54.11 (IS) vs. 3.53% ± 42.67 (CS), *P* = 0.071], whereas the main curve of the two groups was significantly different (*P* = 0.049). The correction rate of compensatory curves was not statistically significant in both the first brace and the last follow-up (P > 0.05) (Table 4).

**Table 4:** Correction rate of the first treatment and last follow-up according to etiology diagnosis.

Radiographic characteristics	IS (13)	CS (14)	*P* value
**Pretreatment, ° (SD)**			
Main Cobb angle	34.92 (10.94)	41.21 (8.60)	0.108
Proximal compensatory curve	12.85 (13.15)	19.85 (9.80)	0.137
Distal compensatory curve	22.43 (10.34)	25.00 (11.82)	0.644
**Correction in first brace**			
Main Cobb angle (deg.)	18.69 (12.06)	33.93 (10.31)	0.002
Main Cobb angle,%(SD)	48.61 (23.66)	17.10 (16.89)	0.000
Proximal compensatory curve,%(SD)	46.61 (30.75)	14.25 (45.34)	0.078
Distal compensatory curve,%(SD)	22.34 (41.24)	1.91 (51.77)	0.393
**Correction of last follow up**			
Main Cobb angle, (deg.)	23.08 (22.15)	37.93 (14.74)	0.049
Main Cobb angle,%(SD)	38.76 (54.11)	3.53 (42.67)	0.071
Proximal compensatory curve,%(SD)	− 0.71 (68.11)	− 13.48 (76.63)	0.692
Distal compensatory curve,%(SD)	27.59 (39.20)	28.53 (34.41)	0.958
Case of success [n (%)]	9 (33.33)	8 (29.63)	–
Improved and follow-up, [n (%)]	6 (22.22)	2 (6.67)	–
Still in brace, [n (%)]	3 (11.1)	6 (22.22)	–
Case of failure, [n (%)]	4 (14.81)	6 (22.22)	–
Operated [n (%)]	2 (7.41)	6 (22.22)	
Still in brace, [n (%)]	2 (7.41)	0	
Duration of brace, months(SD)	79.15 (18.16)	65.21 (20.82)	0.077

IS: Idiopathic scoliosis, CS: Congenital scoliosis.

The main Cobb angle was corrected to 18.69 ± 12.06° (48.61%) after the first bracing and 23.08 ± 22.15°(38.76%)after brace removal at the last follow-up in the IS group. In the CS group, the main Cobb angle was corrected to 33.93 ± 10.31°(17.1%) after the first bracing and 37.93 ± 14.74°(3.53%) after brace removal at the last follow-up. 9 of the 13 patients in the IS group were successful, of which 6 patients had finished bracing and were under follow-up observation, 3 were still in brace, 4 patients in the IS group failed, of which 2 patients had undergone surgery, and 2 were still in brace. In the CS group, 8 of the 14 patients were successful, of which 2 patients achieved resolution and were under follow-up observation, 6 were still in brace, and the other 6 patients who experienced failure had all undergone surgery (Table 4).

## Discussion

To our knowledge, few reports have focused on braces in the treatment of congenital early-onset scoliosis and comparison with progressive idiopathic early-onset scoliosis. Patient demographic characteristics, radiographs, and final outcomes were collected. Age of initial brace and last follow-up, sex, and etiology diagnosis were collected as demographic characteristics, but we did not record their BMI because all the children had good muscle tone and no laxity that would tolerate a delay in treatment and had the ability to resolve reported by Mehta^3^. For the radiograph data, we obtained the compensatory curve of both proximal and distal, and coronal migration of the distance from cervical 7 to the center sacral vertical line (C7-CSVL, mm) in addition to the main curve in the coronal plane to assess scoliosis and balance, in the sagittal plane, thoracic kyphosis, lumbar lordosis and the sagittal vertebral axis (SVA) were measured. Because children with EOS cannot take pulmonary function tests, Glotzbecker^21^ and Johnston et al.^22^ reported that the T1-12 height and the coronal chest width can be used to evaluate the forced expired volume in 1 s (FEV1) and forced vital capacity (FVC). In our study, the T1-12 height increased in both the first brace and the last follow-up. Coronal chest width increased greatly in the last follow-up (*P* = 0.000). These two factors, together with the increase in thoracic kyphosis, indicate that pulmonary function has not been limited by the brace.

We analyzed the series according to Cobb angle correction of the main curve to identify factors that indicate a good response to brace. Patients with a diagnosis of IS and apical vertebra of the main curve located in the thoracolumbar or lumbar region tend to achieve good correction. 6 of the 8 operated patients had thoracic apical vertebra. 8 patients had achieved resolution and stopped the brace for at least 2 years, of which 6 were IS, and they will be followed up closely until maturity assessed by the Risser sign.

Pretreatment RVAD of > 20° was considered to be an important indicator of high risk of progression, we adopted the brace for children with IS who had an RVAD > 20°. Iorio et al.^11^ found that correction differences in radiographic deformity are apparent at longer follow-up, and the amount of correction obtained at initial casting does not confirm treatment success. All the children treated with braces in our study were followed up for at least 4 years (49–117 months). The same outcomes were found as the following: the Cobb angle correction was not significantly different between the success group and failure group at initial bracing (*P* = 0.093), whereas differences in the radiographs were apparent at the last follow-up (*P* = 0.000).

The effects of brace were different according to previous reports. Babaee et al.^23^ reported 27 patients (36%) failed in the brace treatment for juvenile-onset idiopathic scoliosis up to skeletal maturity. Another retrospective review about bracing for juvenile idiopathic scoliosis from bracing to skeletal maturity reported by Whitaker et al.^24^, concluded that Surgery was avoided in 33% of children with minimal to no progression. A prospective study reported by Aulisa et al.^25^ concerning about brace treatment in juvenile idiopathic scoliosis showed that the mean curve magnitude (CM) was 29.6° at initial and 16.9° at last follow up, which was statistically significantly different. Curve correction was accomplished in 88 patients (77.8%), only 4.9% of patients need surgery. In our study, 9/13 IS (69.23%) were succeed, 2/13 IS (15.38%) needed surgery at last follow-up, this may be due to the higher mean value of initial CM in our series (34.92°). Mehta^3^ found that curve resolution occurred in younger children with smaller Cobb angles (average, 32°). Besides, Khoshbin et al.^26^ previously analysed the outcomes of bracing in juvenile idiopathic scoliosis until skeletal maturity or surgery, reported on 50% underwent surgery, and the operative rate was higher for patients with curves 30° or more than those with curves 20° to 29° prior to brace treatment.

Babaee et al.^27^ reported 29 Infantile Idiopathic Scoliosis with the average curve magnitude of 35.62° at the time of diagnosis. Based on their results, brace treatment failed for a total of 20 patients (69%). Of these patients, 12 cases (60%) reached spinal fusion, and four patients (13%) in the surgery-treated group underwent surgery before the age of 10. Smith et al.^28^ reported 17 infantile Idiopathic Scoliosis treated with brace, 9 (52.9%)patients had curve progression and went on to other forms of treatment, 8(47.06%) who did respond, there was an overall improvement of 51.2%. In our study, 7 cases started brace before 3 years old, 3 of them are CS and 4 with IS. Since the number of infantile scoliosis in the two groups was basically the same and small, we did not analyze infantile scoliosis separately. Of the 4 infantile idiopathic scoliosis, no case was operated at the last follow-up.

Wang et al.^29^, confirmed that brace treatment can serve as a time-buying tactic for patients with CS, their study included cases aged younger than 8 years. 9/39 patients underwent surgical intervention, with the time of surgery delayed for 32.1 months. 6/14 patients with CS underwent surgical intervention in our study at the last follow-up, and their average age at surgery was 136 months, the average time delayed by brace was 65 months. Bess et al.^30^ reported a 13% reduction in complication rates for each year of increased patient age at the initiation of instrumentation. Since they are more than 9 years old, surgery can be performed more safely.

The greatest advantage of bracing is that children do not need to be exposed to anesthesia repeatedly, which is associated with increased disability in language and abstract reasoning^31^. There has been growing concern about the detrimental effects of certain anesthetic agents on the developing brain, as indicated by the Food and Drug Administration (FDA)^17^;^32^, while the brace can be implemented in the waking state, so that it deprives the anesthesia risk as well as spinal cord and nerve injury. Moreover, pressure sores on the skin can be avoided because parents can discover them in a timely manner.

In our series, cases were either congenital EOS or progressing idiopathic EOS, which were classified into CS and IS. We found that IS and apical vertebra of the main curve located in the thoracolumbar or lumbar region appeared to be important favorable factors. The majority of Cobb angle correction can be achieved during the first bracing, which may inspire parents to supervise their children while wearing braces. Congenital EOS that did not respond to bracing will be able to delay spinal instrumentation at least. In addition, during the follow-up, bracing was not associated with any complications. With a standardized treatment protocol, we suggest that those with magnitude < 60°, bracing can be an effective and safe strategy in dealing with progressive EOS.

However, the retrospective nature of this study limits the ability to survey patients, and there was no documentation in the clinical notes concerning brace intolerance. In addition, the sample size was small, and our study including cases from June 2011 to July 2023, focused on radiographic outcomes and did not include health-related quality of life (HRQOF) , because The Chinese version of the Early-onset Scoliosis Quality of Life 24-item Questionnaire(EOSQ-24) was available since 2021. In future studies, we may have chance to increase the number of cases and continue to follow the subjects until skeletal maturity and record the HRQOF**.**

## Data Availability

The datasets used and/or analysed during the current study available from the corresponding author on reasonable request.
